# The effect of aerobic exercise with blood flow restriction on substrate utilization and energy expenditure

**DOI:** 10.3389/fspor.2026.1738777

**Published:** 2026-02-20

**Authors:** Jakob D. Lauver, Nathen Andrews, Chase Harris, Nelo E. Zanchi, Kelly E. Johnson, Timothy R. Rotarius, Justin P. Guilkey

**Affiliations:** 1 College of Health, Ball State University, Muncie, IN, United States; 2 Department of Kinesiology, Coastal Carolina University, Conway, SC, United States; 3 Department of Physical Education, Federal University of Maranhao (UFMA), Sao Luis, Brazil

**Keywords:** carbohydrate oxidation, cycling, excess energy expenditure, excess post-exercise oxygen consumption, fat oxidation

## Abstract

**Purpose:**

This study examined the effects of intermittent blood flow restriction (LI-BFR) compared to low- (LI) and high-intensity interval exercise (HI) on substrate utilization and energy expenditure (EE).

**Methods:**

Exercise protocols consisted of 2-minute work intervals interspersed with 1-minute recovery intervals (20W), HI consisted of 5 intervals performed at a workload corresponding to 140% of ventilatory threshold intensity and LI and LI-BFR consisted of 10 intervals performed at 70% of ventilatory threshold. BFR cuffs were inflated to 80% of limb occlusion pressure during each work interval and deflated during each recovery. Following each exercise protocol subjects rested for three hours, during which excess EE and substrate utilization were measured. For all comparisons, statistical significance was set at *p* ≤ 0.05.

**Results:**

Fat oxidation rate was significantly greater in LI-BFR (0.11 ± 0.03 g min^−1^) compared to HI (0.09 ± 0.03 g min^−1^) at 120 MIN (d = 1.13). Fat oxidation in LI-BFR (60 MIN = 0.14 ± 0.01 g·min^−1^, 120 MIN = 0.11 ± 0.01 g min^−1^) was greater than LI (60 MIN = 0.08 ± 0.02 g min^−1^, 120 MIN = 0.9 ± 0.02 g min^−1^). Total excess EE was greater in LI-BFR (184.0 ± 37.6 kcals) compared to HI (127.0 ± 12.0 kcals; d = 3.23) and LI (144.83 ± 35.5 kcals; d = 2.21).

**Conclusion:**

This study suggests that the addition of BFR to low-intensity results in increased fat oxidation following exercise as well as increased EE. The observed increased fat oxidation and EE could potentially have significant long-term effects on weight management.

## Introduction

Aerobic exercise training is known to enhance fat oxidation, a key mechanism supporting metabolic flexibility and energy substrate utilization (1). Impaired fat oxidation, both at rest and during exercise, has been associated with alterations in insulin sensitivity and overall metabolic function (1–3). High-intensity interval training has become popular due to the lower volume of exercise required to achieve positive chronic responses. Previous investigations have shown that high-intensity interval training is a viable alternative to moderate-intensity training to improve fat oxidation and metabolic health (2, 4), yet utilizing such high-intensity workloads may not be well tolerated or appropriate for certain individuals (5). Because high-intensity workloads are often poorly tolerated, there is a need for a paradigm that can deliver similar benefits while operating at lower exercise intensities*.* Several recent studies have found positive chronic responses when utilizing low-intensity exercise combined with blood flow restriction (BFR) (6–8). BFR typically involves the application of a tourniquet or compressive cuff device placed proximally on the exercising limbs that partially restricts blood flow to the exercising musculature (9).

The addition of BFR to aerobic based exercises can improve aerobic capacity, time to exhaustion, the onset of blood lactate accumulation, muscular strength, and markers of metabolic health (7, 9–11). However, these improvements have not been universally observed (12, 13), and may be attributed to different methodological approaches (9). Although the exact mechanism(s) responsible for the observed chronic responses after aerobic training with BFR are not completely understood, the application of BFR alters the local metabolic environment as a consequence of the reduced oxygen availability due to the restricted blood flow (14). Our laboratory (15) and others (6) have demonstrated that the addition of BFR to various low- and moderate-intensity exercises resulted in similar muscle deoxygenation profiles as free-flow, high-intensity interval training. This reduced oxygen availability is indicative of increased local hypoxic and metabolic stress, which may contribute to angiogenesis (10) and mitochondrial biogenesis following aerobic training with BFR (9, 13). Angiogenesis and mitochondrial biogenesis are both associated with greater fat oxidation at rest and during exercise and improved metabolic health (1, 2).

The alterations in the local metabolic environment during the application of BFR may also result in acute alterations in substrate utilization (4), exercise energy expenditure (16–18), and recovery energy expenditure, as measured by excess post-exercise oxygen consumption (EPOC) (17). The hypoxic intramuscular environment during exercise with BFR could increase reliance on glycolytic metabolism which would impact glycogen degradation during exercise, substrate utilization, and energy expenditure during and following exercise (16, 19). Additionally, lower intensity exercise with BFR has been shown to alter neuromuscular recruitment, potentially leading to recruitment of higher threshold motor units, which preferentially prefer glucose (15, 20). This potential increase in carbohydrate oxidation during exercise may alter post-exercise substrate utilization, resulting in greater reliance on fat oxidation during recovery (2, 21). In high-intensity interval training, the elevated energy demand drives an increase in glycolytic flux and glycogen utilization, which has been associated with greater fat oxidation and higher energy expenditure during recovery (21, 22). Similarly, exercise performed under moderate hypoxic conditions has been shown to significantly increase post-exercise lipid oxidation and suppress carbohydrate oxidation for up to 22 h after exercise, suggesting that hypoxia-induced metabolic stress and glycogen depletion may drive this shift toward fat utilization during recovery (23). Exercise with BFR has also been shown to elevate norepinephrine and growth hormone concentrations, which may stimulate lipolysis (24, 25). Therefore, it is possible that the alterations resulting from the application of BFR could also increase post-exercise energy expenditure and fat oxidation as observed in high-intensity interval exercise, albeit at a lower work rate. Although this assertion has yet to be studied and remains unclear, understanding the acute post-exercise responses to BFR could provide insight into the physiological mechanisms underlying substrate utilization and energy metabolism following exercise.

The primary aim of this investigation was to examine the effects of low-intensity interval cycling with BFR compared to low- and high-intensity interval cycling on substrate utilization and energy expenditure. We hypothesized that following the low-intensity BFR and high-intensity conditions fat oxidation would be greater compared to the low-intensity condition (free flow). Secondly, we hypothesize that EPOC would be greater following low-intensity BFR and high-intensity compared to the low-intensity condition. Third, we hypothesized that total energy expenditure would be greater in the low-intensity BFR condition compared to either the low-intensity or high-intensity conditions.

## Methods

### Experimental approach to the problem

Participants were required to report to the laboratory on four separate visits. During the first visit, anthropometric measurements were taken along with the estimation of daily total energy expenditure and the assessment of peak exercise responses. The second, third, and fourth visits served as experimental conditions. The experimental conditions used an experimental design with a repeated-measures statistical plan, in which each participant performed all three experimental conditions: Low-intensity (LI), Low-intensity with BFR (LI-BFR), and High-intensity (HI). Each experimental condition consisted of three phases: pre-exercise resting phase, exercise phase, and recovery phase. All conditions were the same except for the exercise protocol.

### Participants

Participants were informed of the risks and benefits of the study, prior to participation, and written informed consent was obtained from all individuals participating in the study. The study complied with the standards set by the Declaration of Helsinki and was approved by the University's Institutional Review Board (IRB #: 2020.38). All participants recruited for this study had no diagnosed cardiovascular, pulmonary, or metabolic disease, and were not taking medications that influenced cardiovascular responses, based on their responses to a health history questionnaire. Participants were excluded if they self-reported a history of metabolic, pulmonary, cardiovascular disease, or an orthopedic-related injury in the past 6 months. In addition, participants were asked to refrain from any unaccustomed strenuous physical activity, maintain their normal dietary habits, and not to take any anti-inflammatory drugs or nutritional supplements during the experimental period.

There were 11 participants recruited for this study; however, ten of the participants were included in the analysis. One participant's data was excluded due to an equipment error during one of their visits. Power calculations were performed using effect sizes from a previous study that examined EPOC and fat oxidation with interval exercise (26). The most conservative effect size (d = 0.6) indicated that a sample size of 7 subjects would be sufficient what *α* = 0.05 and *β* = 0.80. The sample size recruited in this study is comparable to other studies examining acute protocols with a crossover design (26, 27). Among the ten participants in the study, there were seven males and three females. Participant characteristics are shown in Table 1. The total work output was 2,421.6 ± 567.6 W in each exercise protocol. The equal total work outputs were part of the design of the study.

**Table 1:** Participant characteristics. Data is represented as means ± SD.

N (sex)	Age (yrs)	Height (cm)	Weight (kg)	Body Fat (%)	VO_2_peak (L·min^−1^)	VO_2_peak (mL·kg^−1^·min^−1^)	WRpeak (W)
10 (♂ = 7; ♀ = 3)	25.1 ± 6.0	172.4 ± 4.1	75.8 ± 12.9	21.8 ± 7.5	2.74 ± 0.82	36.0 ± 7.5	252.3 ± 59.1

### Procedures

#### Visit 1

Upon arrival to the laboratory on the first visit, body composition was assessed by whole-body densitometry using air displacement plethysmography (Bod Pod®, Cosmed, Concord, CA USA). Prior to the 1st visit, participants were instructed to refrain from strenuous activity for 12 h, refrain from alcohol consumption for 12 h, maintain adequate hydration, and instructed to eat a light meal in preparation for the graded exercise test. Participants then performed a graded exercise test (GXT) on an electronically braked cycle ergometer (Corival, Lode, Groningen, Netherlands) to determine peak exercise responses. The GXT commenced at 20 W for four minutes followed by a ramped 20 W·min^−1^ increase in work rate (WR) until volitional exhaustion. Pulmonary gas exchange (V˙O_2_, V˙CO_2_) and minute ventilation (V˙_E_) were measured breath-by-breath throughout the exercise protocol using a commercially available metabolic measurement system that that was calibrated prior to each trial according to manufacturer recommendations (Quark CPET, COSMED, Rome, Italy) and averaged into 10-s intervals. Expired gas flows were measured using a turbine and optoelectronic reader that was calibrated prior to each trial using a syringe with a known volume (3.0 L). The O_2_ and CO_2_ analyzers were also calibrated prior to each trial using precision analyzed gases with known concentrations. Corrections for the ambient temperature and relative humidity were made for the conditions measured near the subject's mouth. The COSMED Quark CPET demonstrated excellent validity compared to the Douglas bag method, with VO₂ and VCO₂ correlations of r ≈ 0.99 and mean differences <2% (28, 29), and strong reliability across days with VO₂ variability within ±4%.

The GXT was terminated when participants could no longer maintain a pedal cadence greater than 40 rpm. The highest volume of oxygen consumed (VO_2_) averaged over a 10-second interval was taken as peak oxygen consumption (VO_2peak_). The ventilatory threshold (VT) was estimated by visual inspection from gas exchange indices (30) and confirmed by determining the VO_2_ at which there was an increase in the ventilatory equivalent for VO_2_ (VE/VO_2_) and end-tidal PO_2_ with no concomitant increase in the ventilatory equivalent for VCO_2_ (VE/VCO_2_) or end-tidal PCO_2_. Ventilatory thresholds were validated by two co-authors, who were each blinded to the others' interpretation.

### Pre-Exercise experimental protocol

The experimental conditions were performed on different days separated by at least 48 h and assigned in a randomized order (http://www.randomization.com). Each participant performed the experimental conditions at approximately the same time in the morning. Environmental conditions of the experimental visits are shown in Table 2. Three hours prior to each experimental condition, participants were asked to consume a standardized meal, based on an estimation of their total energy expenditure. For the purposes of the standardized meal, total estimated energy expenditure consisted of estimations of resting energy expenditure via the Nelson equation (31, 32) and estimation of daily physical activity energy expenditure, as determined by responses to the International Physical Activity Questionnaire (IPAQ) (33). The total caloric intake of the standardized meals was approximately 15%–20% of the participant's total estimated energy expenditure. This percentage is equivalent to the percentage of calories consumed by an average adult during breakfast (34). The standardized meal consisted of a nutritional shake (Ensure® Original Nutrition Shake, 220 calories per serving, 33 g carbohydrates, 6 g fat, and 9 g protein) and a cereal bar (Kroger® Fruit & Grain Cereal Bar, 130 calories per serving, 25 g of carbohydrates, 3 g of fat, and 1 g of protein). The number of servings of the nutritional shake and cereal bars were adjusted to ensure each participant consumed between 15% and 20% of their estimated total energy expenditure. The mean calories consumed in the standardized meal was 434.0 ± 68.0 kcals (76.3 ± 12.2 g carbohydrates, 11.4 ± 1.8 g of fat, 11.5 ± 2.5 g of protein). In addition to consuming the standardized meal, participants were asked to avoid consumption of caffeine, alcohol, and tobacco and avoid vigorous activity for at least 12 h and 24 h prior to each experimental condition, respectively. Adherence to all pre-condition requirements, including consuming the entire assigned meal, were verbally confirmed by the investigator prior to the start of the experimental conditions.

**Table 2:** Temperature, barometric pressure, and humidity during each experimental condition.

Condition	Temperature (C)	Barometric Pressure (mmHg)	Humidity (%)
BFR	22.0 ± 0.0	760.9 ± 3.0	45.9 ± 10.0
HIE	22.0 ± 0.0	763.7 ± 5.3	46.2 ± 12.4
LIE	21.9 ± 0.3	763.2 ± 5.8	46.8 ± 14.0

In order to control for daily variations in resting VO_2_, the participants were asked to rest in a semi-recumbent position for 30 min for each experimental condition. After the first 15 min of the resting period, the participants were fitted with a facemask, and breath-by-breath gas exchange and heart rate (HR), via a chest strap HR monitor, were continuously collected for the final 15 min of the resting period.

### Exercise protocols

The exercise protocols were matched for total work output and were performed on an electronically-braked cycle ergometer (Corival, Lode, Netherlands). Participants were instructed to maintain a pedal rate of 60–80 RPM throughout the protocol. Each exercise protocol started with a four-minute warm-up (20W). For the LI-BFR and LI exercise conditions, participants completed 10 two-minute work intervals interspersed with one-minute recovery. Work intervals were performed at a cycling work rate that elicited a VO_2_ corresponding to 70% of VT, as determined from the GXT. The workload for the recovery intervals was 20 W. During the LI-BFR condition, the BFR cuffs (SC10D, Hokanson, Bellevue, WA, 10.0 cm width) were placed proximally on both legs and inflated to 80% of limb occlusion pressure (LOP) at the start of the work intervals and remained inflated throughout each work interval. While the degree of restriction has been shown to effect cardiovascular responses (35), at limb occlusion pressures above 60%, muscle responses become pressure-dependent, with local muscle oxygenation decreasing and neuromuscular activation and blood lactate increasing during low-intensity exercise (36–38)., Therefore, 80% LOP was used throughout each occlusion condition, which is in line with previous research (15). During all recovery intervals, the cuffs were rapidly deflated and remained deflated until the next work interval. For the HI, participants completed five two-minute work intervals interspersed with one-minute recovery. Work intervals were performed at a work rate corresponding to 140% of VT, as determined from the GXT. This work:rest ratio was utilized so that the total work performed during all conditions was equal. During all recovery intervals, the power output was reduced to 20 W. Breath-by-breath gas exchange and HR, via a chest strap HR monitor, were continuously collected throughout each exercise protocol. Baseline VO_2_ was considered the average VO_2_ over the final 10 min of rest.

### Recovery protocol

Immediately following each exercise protocol, participants assumed a semi-recumbent position and were asked to remain seated for the 3-hour recovery period in order to measure EPOC and substrate utilization. Immediately at the start of recovery, breath-by-breath gas exchange was collected continuously for 30 min, except for a short break at 15 min for the participant to drink water. Following the first 30 min, gas exchange was collected for 8 min of every 15-min period between 30 and 180 min. While the gas exchange was not being collected the participants could drink water ad-libitum while remaining in the semi-recumbent position. HR was continuously collected over the entire recovery period. Participants were allowed two breaks from the semi-recumbent position at 60 min and 120 min to use the bathroom, if necessary.

### BFR application

To determine the BFR pressure for each participant, cuffs (SC10D, Hokanson, Bellevue, WA, 10.0 cm width) were placed around the proximal portion of both thighs. Participants laid supine on a treatment table and rested for five minutes. Then the posterior tibial artery was identified on the participant's dominant leg using Doppler auscultation (Nicolet Imex Pocket Dop II, Natus, San Carlos, CA). Once the pulse was identified, the cuffs were rapidly inflated to 50mmHg and then progressively inflated until the pulse was eliminated. The pressure associated with the cessation of the pulse was taken as the limb occlusion pressure (LOP) (6). This procedure was performed prior to all exercise protocols that involved the application of BFR. During all protocols that involved BFR, the cuffs were inflated to each participants' custom pressure based on their LOP (80% LOP) (15).

### EPOC

The magnitude of EPOC was calculated over the first 90 min of recovery. For each condition, the absolute VO_2_ (L·min^−1^) was averaged over the final 10 min of the resting period and was considered the baseline measurement for that condition. During recovery, VO_2_ was averaged over 15-s during the following times: 0–15 min, 17–30 min, 38–45 min, 53–60 min, 68–75 min, and 83–90 min. For each 15-s VO_2_, a netVO_2_ was calculated by subtracting the baseline VO_2_ from the 15-s VO_2_. The netVO_2_ was then plotted against the time during recovery and EPOC was calculated as the area under the curve via the trapezoidal rule.

### Excess energy expenditure

During each condition breath-by-breath VO_2_ (L·min^−1^) averaged every 15 s from the start of the first work interval to the end of the last work interval. For each 15-s VO_2_, a netVO_2_ was calculated by subtracting the baseline VO_2_ from the 15-s VO_2_. Exercise excess energy expenditure and recovery excess energy expenditure were calculated from the netVO_2_ (L·min^−1^) during exercise and the first 90 min of recovery, respectively. Due to limitations of using RER-based caloric equivalents during high-intensity exercise, net VO₂ (L·min^−1^) was multiplied by a constant factor of 5 kcal·L^−1^ O₂ for all conditions (pre-exercise, exercise, and recovery) to estimate energy expenditure. The exercise and recovery excess energy expenditures were summed to calculate the total excess energy expenditure.

### Substrate oxidation calculations

Fat oxidation rates and carbohydrate oxidation rates were calculated from the average VO_2_ and VCO_2_ from the final 10 min of rest and 10-minute averages during recovery between 50 and 60 min (60 MIN), 110–120 min (120 MIN), and 170–180 min (180 MIN). Calculating fat oxidation and carbohydrate oxidation by indirect calorimetry assumes steady-state conditions which may not be present during the first 60 min of recovery from high-intensity exercise. Bicarbonate buffering and non-metabolic CO_2_ have been shown to be no different from resting control conditions from 60 to 120 min after high-intensity exercise (39). Therefore, exercise and data early in recovery (first 50 min of recovery) were excluded from the analysis. Fat and carbohydrate oxidation rates were calculated based on equations developed by Peronnet and Massicotte (40). The equations were as follows:

$$\begin{array}{c} \mathrm{Fat}\;\mathrm{oxidation}\;\mathrm{rate}\;(g\cdot \mathrm{mi}n^{\text{-}1})\;=\;1.695\cdot VO_{2}\text{–}1.701\cdot \mathrm{VC}O_{2}\\ \mathrm{Carbohydrate}\;\mathrm{oxidation}\;\mathrm{rate}\;(g\cdot \mathrm{mi}n^{\text{-}1})\;=\;4.585\cdot \mathrm{VC}O_{2}\text{–}3.226\cdot VO_{2} \end{array}$$

### Statistical analysis

Statistical analyses were completed using IBM SPSS statistical software (Version 25.0; SPSS, Inc., Chicago, IL). Shapiro–Wilk test was conducted prior to all ANOVA analyses to assess the normality of the data. The test was non-significant in all cases, indicating no violations of normality for any variable. Additionally, test-retest reliability during rest was assessed using the intraclass correlation coefficient (ICC). A two-way mixed-effects model with absolute agreement was used, and ICC was calculated for average measures, reflecting the reliability of the mean measurement across conditions. ICC values were interpreted as: <0.50 = poor, 0.50–0.75 = moderate, 0.75–0.90 = good, and >0.90 = excellent reliability.

A two-way (condition [LI-BFR, HI, LI] by time [Rest, Ex, 60 MIN, 120 MIN, 180 MIN) repeated measures ANOVA was used to compare the absolute VO_2_ (L·min^−1^), relative VO_2_ (mL·kg^−1^·min^−1^), RER, and HR. A two-way (condition [LI-BFR, HI, LI] by time [Rest, 60 MIN, 120 MIN, 180 MIN) repeated measures ANOVA was used to compare the fat oxidation rate and carbohydrate oxidation rates. A two-way (condition [LI-BFR, HI, LI] by time [exercise, recovery) repeated measures ANOVA was used to compare exercise and recovery excess energy expenditure between conditions. A one-way repeated measures ANOVA was used to compare total excess energy expenditure and EPOC between conditions. Subsequent Bonferroni pairwise *post-hoc* comparisons were made when necessary. Cohen's f and d were used as an estimate of effect size for the ANOVA and subsequent *post-hoc* comparisons, respectively. Cohen's f values were interpreted as follows: a small effect was defined as <0.25, a medium effect as 0.25–0.40, and a large effect as >0.40. Cohen's d values were interpreted as follows: a small effect was defined as <0.40, a medium effect as 0.40–0.75, a large effect as 0.75–1.10, a very large effect as 1.10–1.45, and a huge effect as >1.45 (41). Statistical significance was established if *p* ≤ 0.05.

## Results

Mean VO_2_ at VT was 1.58 ± 0.39 L·min^−1^. As such, the WRs associated with the exercise protocols for 70% VT and 140% were 68.3 ± 23.1 and 136.5 ± 46.3 W, respectively. The WRs during work intervals were significantly different by design of the study. The total work output was 2,421.6 ± 567.6 W in each exercise protocol. The equal total work outputs were part of the design of the study.

### Physiological Responses

The physiological responses (absolute VO_2_ (L·min^−1^), relative VO_2_ (mL·kg^−1^·min^−1^), VCO_2_ (L·min^−1^), RER, and HR during each condition are shown in Table 3. Absolute VO_2_ (L·min^−1^), relative VO_2_ (mL·kg^−1^·min^−1^), VCO_2_ (L·min^−1^), and RER did not meet the assumption of sphericity, so a Greenhouse-Gasser test was performed. There was a significant condition by time interaction for absolute VO_2_ (L·min^−1^) (*p* < 0.001; f = 2.64), relative VO_2_ (mL·kg^−1^·min^−1^) (*p* < 0.001; f = 2.22;), VCO_2_ (L·min^−1^) (*p* < 0.001; f = 1.93), RER (*p* < 0.001; f = 1.38;), and HR (*p* < 0.001; f = 1.89). The decomposition of the interaction showed that while pre-exercise VO_2_ (absolute or relative) did not differ between conditions (resting ICC = .900 and.912, respectively), during exercise, LIBFR was significantly higher compared to HI and LI (for all comparisons; 0.61 ≤ d ≤ 1.86). It then decreased after 60 min of recovery, only to become significantly higher again at the 120-minute recovery period compared to HI (d = 0.84 and d = 0.75, respectively) and LI (d = 1.07 and d = 0.87, respectively). Note that HI was also significantly higher compared to LI during exercise. The difference between HI and LI at 120 MIN were not significant. There were no differences between conditions for absolute VO_2_ (L·min^−1^) and relative VO_2_ (mL·kg^−1^·min^−1^) at 60 MIN and 180 MIN.

**Table 3:** Physiological responses during rest prior to exercise, exercise, and 60, 120, and 180 after exercise (*N* = 10).

Time	VO_2_ (L·min^−1^)			VO_2_ (mL·kg^−1^·min^−1^)			VCO_2_ (L·min^−1^)			RER			HR (bpm)		
	LI-BFR	HI	LI	LI-BFR	HI	LI	LI-BFR	HI	LI	HI	LI	LI	LI-BFR	HI	LI
Rest	0.31	0.31	0.31	4.3	4.3	4.4	0.28	0.29	0.29	0.91	0.93	0.92	71	68	72
	±0.04	±0.03	±0.05	±0.6	±0.5	±0.7	±0.03	±0.03	±0.04	±0.04	±0.04	±0.04	±13	±12	±13
Exercise	1.26^†^^‡^	1.60^†^	1.09	17.3^†^^‡^	21.7^†^	15.1	1.25^†^^‡^	1.71^†^	1.40	1.00^†^^‡^	1.10^†^	0.97	141^†^	144^†^	111
	±0.24	±0.34	±0.22	±2.6	±4.2	±2.6	±0.24	±0.45	±0.21	±0.02	±0.03	±0.02	±19	±16	±13
60 MIN	0.33	0.33	0.31	4.5	4.6	4.3	0.26	0.26	0.27	0.80^†^^‡^	0.83	0.85	77^†^	78^†^	67
	±0.04	±0.05	±0.04	±0.7	±0.7	±0.6	±0.03	±0.03	±0.04	±0.04	±0.05	±0.04	±13	±10	±12
120 MIN	0.35^†^^‡^	0.32	0.31	4.9^†^^‡^	4.4	4.3	0.28^†^	0.26	0.26	0.81	0.82	0.83	69^†^^‡^	65	63
	±0.05	±0.04	±0.03	±0.7	±0.5	±0.7	±0.03	±0.03	±0.03	±0.05	±0.06	±0.02	±12	±12	±10
180 MIN	0.34	0.33	0.32	4.7	4.5	4.4	0.27	0.27	0.26	0.82	0.84	0.82	69^†^	66	64
	±0.04	±0.04	±0.04	±0.5	±0.7	±0.5	±0.02	±0.03	±0.03	±0.02	±0.05	±0.02	±12	±10	±11

† significantly different than LI (*p* < 0.05).

‡ significantly different than HI (*p* < 0.05).

Furthermore, VCO_2_ (L·min^−1^) at rest was not different between conditions (ICC = 0.879). During exercise, VCO₂ (L·min^−1^) was higher in HIE than BFR (*p* = .003, d = 1.51) and LIE (*p* < .001, d = 2.12), and higher in BFR than LIE (*p* < .001, d = 2.27). At 120-min recovery, VCO₂ VCO_2_ (L·min^−1^) was higher in BFR than LIE (*p* = .029, d = 1.04), but similar to HIE (*p* = 0.083). The difference in VCO_2_ (L·min^−1^) at 120-recovery between HIE and LIE was not significant (*p* = 1.00). No other pairwise differences were significant at 60-min, or 180-min recovery.

The RER at rest was not different between conditions, but the RER during exercise was significantly different between all conditions (for all comparisons; 1.14 ≤ d ≤ 5.03). The only difference in RER between conditions during recovery occurred at 60 MIN. Specifically, the RER during LI-BFR was significantly lower than the RER during HI (d = 0.69) and LI (d = 0.40).

There were no differences between groups for HR at rest. There was a significantly greater HR during exercise in LI-BFR and HI compared to LI (d = 1.83 and d = 1.82, respectively); the difference between LI-BFR and HI was not significant. During recovery, HR at 120 MIN in LI- BFR was significantly greater than HI (d = 0.38); HR at 60 MIN and 180 MIN were similar between LI-BFR and HI. Additionally, HR during LI-BFR was significantly greater than LI at all points during recovery (60 MIN; d = 0.90; 120 MIN; d = 0.57; 180 MIN; d = 0.48). The only difference in HR during recovery between HI and LI occurred at 60 MIN (d = 0.95).

Descriptive values for ventilation (VE), expired fraction of oxygen (FeO₂), and expired fraction of carbon dioxide (FeCO₂) are presented in Table 4. These variables provide additional physiological context for the metabolic responses across conditions and time points. Because these measures were not part of the primary research purpose, no statistics were performed on VE, FeO₂, or FeCO₂, and they are reported descriptively only.

**Table 4:** Ventilation and expired gas fractions during rest prior to exercise, exercise, and 60, 120, and 180 after exercise (*N* = 10).

Time	Ve (L*min^-1^)			FeO_2_ (%)			FeCO_2_ (%)		
	LI-BFR	HI	LI	LI-BFR	HI	LI	LI-BFR	HI	LI
Rest	10.6	11.5	10.9	17.38	17.41	17.41	3.26	3.31	3.27
	±1.6	±0.8	±1.7	±0.32	±0.45	±0.45	±0.38	±0.37	±0.40
Exercise	44.8	53.5	32.0	17.00	16.75	16.75	3.49	4.16	4.07
	±8.9	±12.2	±4.6	±0.46	±0.52	±0.52	±0.43	±0.51	±0.53
1 HR	10.7	11.1	9.9	17.29	17.07	17.07	2.99	3.12	3.36
	±1.1	±1.3	±1.7	±0.42	±0.43	±0.43	±0.27	±0.45	±0.38
2 HR	11.3	10.1	9.7	17.22	17.09	17.09	3.09	3.18	3.32
	±1.2	±1.1	±1.8	±0.29	±0.48	±0.48	±0.34	±0.36	±0.46
3 HR	10.3	10.3	9.7	17.04	16.98	16.98	3.29	3.25	3.35
	±1.2	±1.1	±1.9	±0.35	±0.58	±0.58	±0.45	±0.36	±0.52

### Fat and carbohydrate oxidation rate

**Figure 1:**
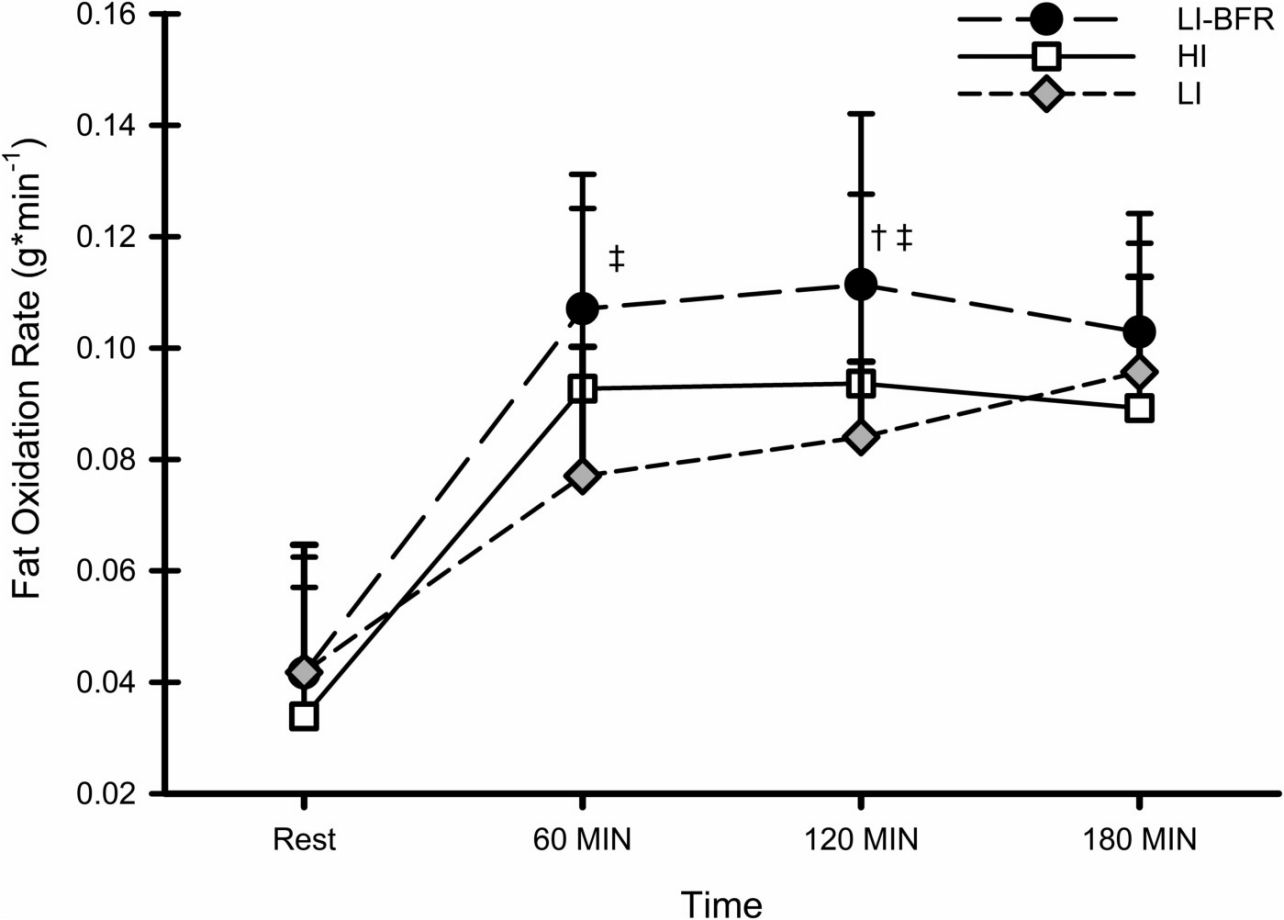
Fat oxidation rates at rest and during recovery. †- significantly different than LI, ‡- significantly different than HI. *N* = 10. Data are presented as mean ± SD.

Fat oxidation rates at rest and during recovery for each condition are shown in Figure 1. There was a significant condition by time interaction for fat oxidation rate (*p* = 0.006; f = 0.62,). The decomposition of the interaction revealed that fat oxidation rate was similar at rest between conditions (ICC = 0.590); however, fat oxidation rate was significantly greater in LI-BFR compared to HI at 120 MIN (*p* = 0.002; d = 1.13). There were no other differences in fat oxidation rates between LI-BFR and HI; the difference at 60 MIN approached, but did not reach significance (*p* = 0.065; d = 0.92). Additionally, fat oxidation was significantly greater in LI-BFR compared to LI at 60 MIN (*p* = 0.015; d = 1.93) and 120 MIN (*p* = 0.002; d = 1.70;); the difference at 180 MIN was not significant. Fat oxidation rate was similar between HI and LI during all points in recovery. Within all conditions, fat oxidation rate during rest was significantly lower than all points during recovery (for all comparisons; *p* < 0.001; 3.56 ≤ d ≤ 5.70).

**Figure 2:**
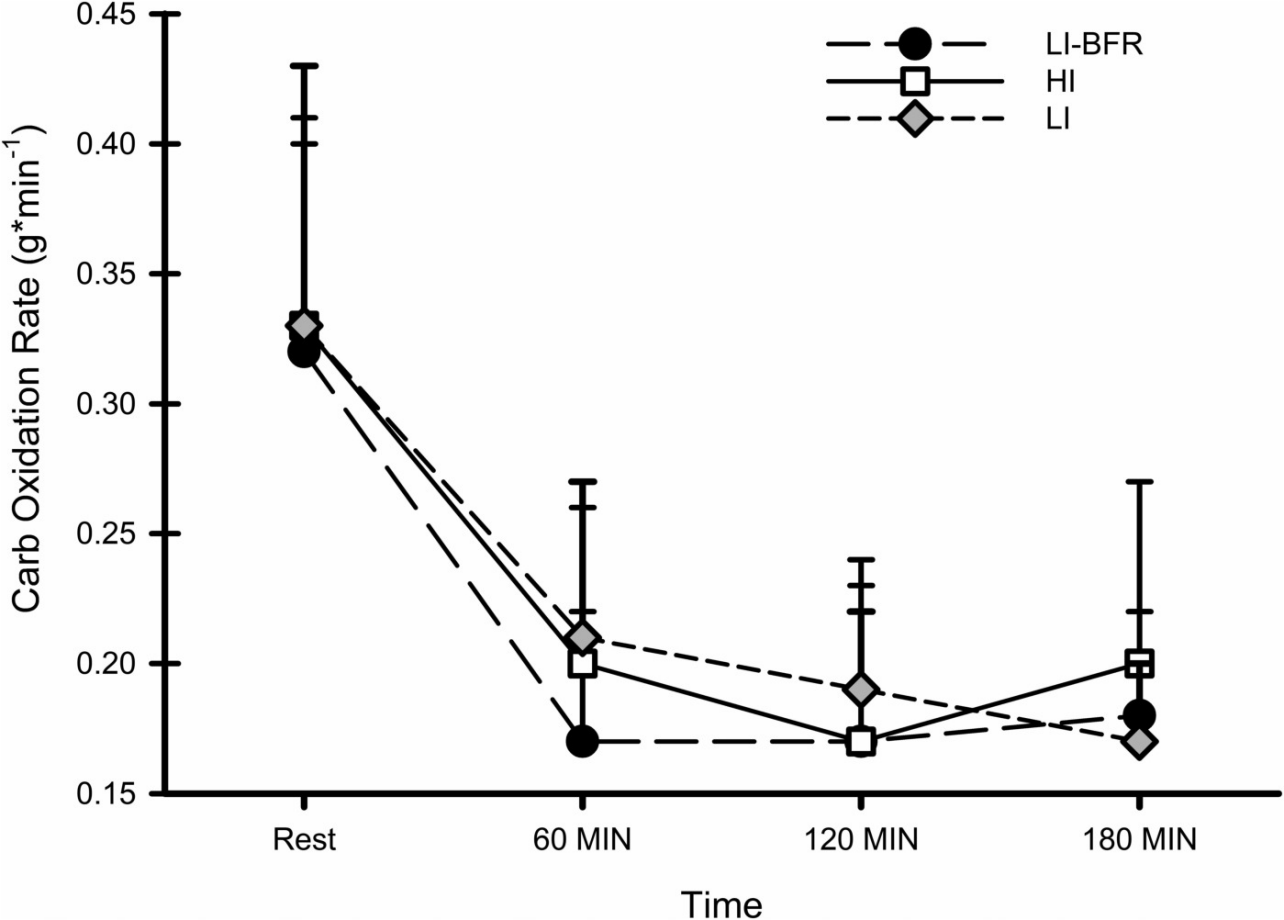
Carbohydrate oxidation rates at rest and during recovery. *N* = 10. Data are presented as mean ± SD.

Carbohydrate oxidation rate responses at rest and during recovery for each condition are shown in Figure 2. The condition by time interaction and the main effect of condition were not significant (*p* *=* *0.623*; resting ICC = 0.711*)*. There was a significant main effect of time (*p* < 0.001; f = 2.42). Specifically, the carbohydrate oxidation rate was significantly greater during rest than at all points during recovery (*p* < 0.001).

### EPOC

**Figure 3:**
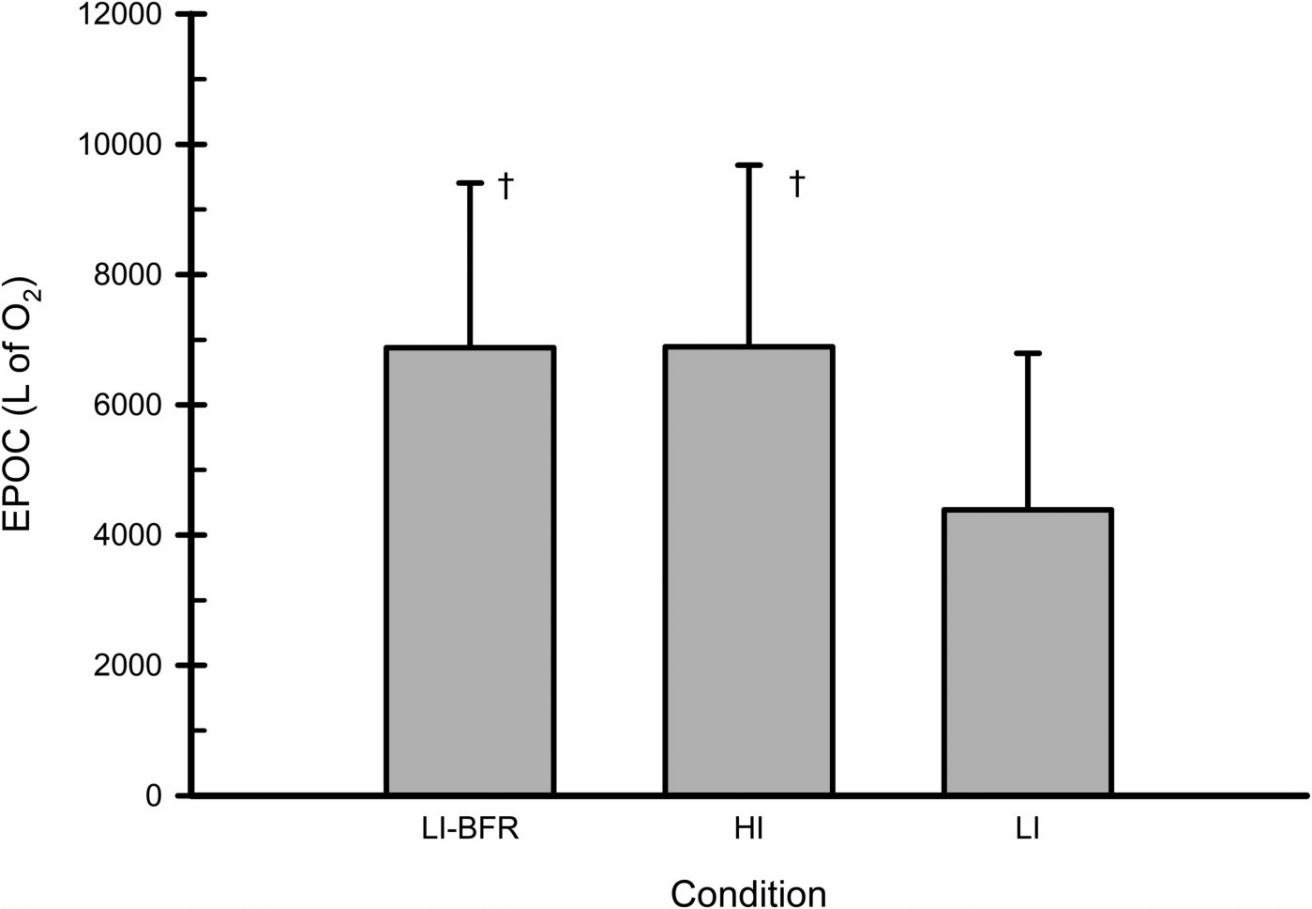
Excess post-exercise oxygen consumption (EPOC) in response to LI-BFR, HI, and LI. †- significantly different than LI. *N* = 10. Data are presented as mean ± SD.

The magnitude of EPOC in each condition is shown in Figure 3. The one-way ANOVA showed a significant main effect (*p* = 0.011; f = 0.85). The magnitude of EPOC in the LI-BFR condition was similar to HI; LI-BFR and HI produced greater EPOC compared to LI (d = 0.97 and d = 0.97, respectively).

### Energy expenditure

**Figure 4:**
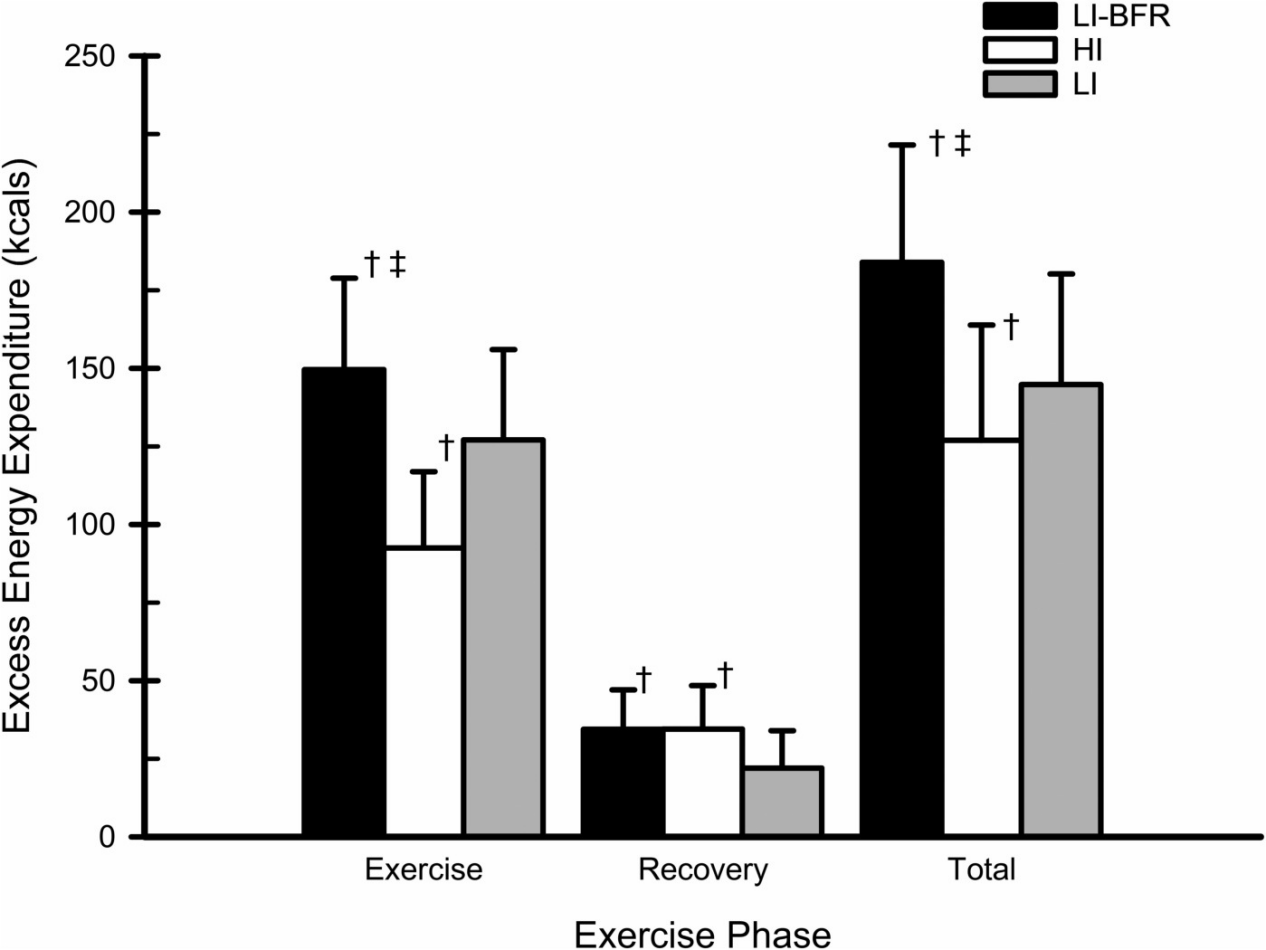
Excess energy expenditure (EE) in response to LI-BFR, HI, and LI. †- significantly different than LI, ‡- significantly different than HI. *N* = 10. Data are presented as mean ± SD.

Exercise, recovery, and total excess energy expenditure in LI-BFR, HI, and LI are shown in Figure 4. There was a significant condition by time interaction for exercise and recovery energy expenditure (*p* < 0.001; f = 3.32). One-way ANOVAs showed a significant effect for total excess energy expenditure (*p* < 0.001; f = 2.62). The decomposition of the interaction revealed a greater excess energy expenditure during exercise in LI-BFR compared to HI (*p* < 0.001; d = 3.58) and LI (*p* < 0.001; d = 1.41). Additionally, LI produced greater excess energy expenditure during exercise compared to HI (*p* < 0.001; d = 2.17). During recovery, there was no difference in excess energy expenditure between LI-BFR and HI, but both were different from LI ((*p* = 0.027; d = 1.68 and (*p* = 0.016; d = 1.68, respectively). One-way ANOVAs showed a significant effect for total excess energy expenditure (*p* < 0.001; f = 2.62). LI-BFR produced the greatest total excess energy expenditure (LI-BFR vs. HI: *p* < 0.001; d = 3.23; LI-BFR vs. LI: *p* < 0.001; d = 2.21). Total excess energy expenditure was also different between HI and LI (*p* = 0.027; d = 1.01).

## Discussion

This investigation aimed to compare substrate utilization and energy expenditure during LI-BFR, LI, and HI cycling. The results showed that fat oxidation during recovery was higher after LI-BFR than LI and HI, supporting our first hypothesis. Both LI-BFR and HI had greater excess post-exercise oxygen consumption (EPOC) than LI, confirming our second hypothesis. Additionally, LI-BFR had the highest total excess energy expenditure, confirming our third hypothesis.

In line with prior research (2, 22, 26), our findings show a post-exercise shift toward fat oxidation, likely driven by the body's prioritization of glycogen resynthesis (42, 43). Exercise under arterial occlusion significantly depletes glycogen in both Type I and II fibers (44). Muscle glycogen depletion through exercise leads to an increased post-exercise glucose uptake response (45), which is mediated by increased GLUT-4 vesicle translocation from the intracellular pool to the muscle plasma membrane (46). Hypoxia may further amplify this response (47), suggesting that the increased fat oxidation response observed in this investigation may be part of a mechanism to spare glucose for muscle glycogen resynthesis.

In the current investigation, higher fat oxidation rates were observed in the LI-BFR condition compared to the LI condition following exercise, which were performed at the same intensity and duration. Recovery fat oxidation rates did not differ between HI and LI, consistent with prior studies showing similar post-exercise fat oxidation following both continuous low intensity and high-intensity interval exercise (21, 43, 48), however, differences have been observed following light-intensity continuous exercise and supramaximal intensities (26, 27). It is thought that the greater shift towards fat oxidation during recovery could be a result of greater glycogen depletion and/or recruitment of Type II fibers during higher intensity exercise (22). In the present study, observed differences in fat oxidation may also be due to the increased reliance on carbohydrate oxidation (muscle glycogen) during the BFR condition. The reduction in arterial inflow associated with the addition of BFR may have caused a preferential shift to more glycolytic metabolism, due to the hypoxic intramuscular environment (15, 16). Additionally, the BFR may have altered muscle excitation and/or muscle recruitment (15, 49). An increase in muscle excitation may be associated with increased recruitment of higher threshold motor units containing more Type II fibers, which are primarily glycolytic. Type II muscle fibers rely more heavily on glycogenolysis for energy production, whereas Type I fibers, due to their higher oxidative capacity, preferentially utilize the oxidative pathway for energy production (50). Although not measured in the present study, the hypoxic environment and greater motor unit recruitment could have increased reliance on carbohydrate oxidation and/or glycolytic metabolism during LI-BFR, which could explain the greater fat oxidation during recovery compared to the LI without BFR.

Additionally, catecholamine- and hormone-induced increases in lipolysis have been proposed mechanisms for increased fat oxidation during recovery from high-intensity exercise (22). Fat oxidation is influenced by free fatty acid availability, which is largely affected by hormone sensitive lipase activity (48). Growth hormone exerts its metabolic effect by stimulating lipolysis, which results in an increased flux of free fatty acids into circulation (51). Exercise with BFR has also been shown to result in an increase in plasma concentrations of growth hormone and norepinephrine during exercise (24). Though not assessed in the current investigation, the potential increase in growth hormone and/or norepinephrine concentrations could have stimulated an increase in lipolysis during BFR which may have contributed to the observed increased fat oxidation rates. Additionally, fat oxidation rates can be affected by the duration and intensity of exercise (48). Another possible contributing factor to the difference in fat oxidation between LI-BFR and HI was the observed differences in energy expenditure during exercise. While HI elicited a higher VO₂ compared to LI and LI-BFR, the longer duration of exercise in the LI and LI-BFR conditions resulted in greater overall energy expenditure during the exercise bout. Previous studies have shown no differences in fat oxidation during recovery following isoenergetic bouts of various intensities (21, 43, 52). Although the total work was controlled within the exercise protocols in the current investigation, the differences in energy expenditure during exercise could have contributed to the differences in fat oxidation.

In regards to our second hypothesis, it has previously been observed that walking with BFR resulted in a greater EPOC magnitude than walking without BFR at the same intensity (17). The current investigation also demonstrated that EPOC following LI-BFR was similar to HI. Exercise intensity and metabolic stress are the primary factors in the magnitude of EPOC, such that EPOC is greater following high-intensity exercise compared to low-intensity exercise (22). Greater EPOC has been associated with lactate-glycogen interconversions, replenishment of oxygen stores, resynthesis of ATP and creatine phosphate, and increased ventilation amongst other factors. Several of these factors have been shown to be affected by the application of BFR despite a lower work rate (53) and may contribute to the observed results in the current investigation. The addition of BFR has also been associated with an increase in blood lactate concentrations, though this has not been universally observed (6, 13). Additionally, we have demonstrated previously that cycling with BFR results in a greater decrease in tissue oxygen saturation compared to low-intensity cycling without BFR (15). The reduced tissue oxygen saturation that occurs with the application of BFR may be indicative of increased glycolytic stress during exercise, contributing to the increase in EPOC observed in the current investigation. These observed differences in EPOC have practical implications for weight loss as EPOC has been attributed as a key component for observed weight loss during low-volume high-intensity interval training (54).

Regarding our third hypothesis, exercise excess energy expenditure and total excess energy expenditure were greater in the LI-BFR condition compared to both the LI and HI conditions. These findings agree with previous investigations that have demonstrated that the addition of BFR to low-intensity exercise resulted in an increase in VO_2_ during exercise at the same intensity (13). This greater VO_2_ observed in the current investigation may potentially be due to a combination of increased cardiorespiratory work or alterations in muscle recruitment. In the current investigation HR during LI-BFR was greater than the LI, but similar to HI. This suggests that there was an increased cardiac stress during exercise. Although not assessed in the current investigation, it has been previously demonstrated that the application of BFR during exercise can result in altered muscle excitation (15). During exercise with BFR, there is an increased metabolic stress, which can lead to earlier-than-normal fatigue of lower threshold motor units, resulting in recruitment of higher threshold motor units in order to maintain the desired force output (20). In the current investigation, the power output during exercise (work and rest intervals) was controlled by an electronically-braked cycle ergometer. Therefore, the power output needed to be continuously met, which may have resulted in the recruitment of higher threshold motor units and contributed to the increased VO_2_ observed during exercise. Additionally, given the dynamic task, the possibility of altered contributions of multiple muscle groups may have contributed to the increased VO_2_.

The current investigation is not without its limitations. First, the comparisons made between BFR and free-flow conditions were made at only one restriction pressure (80% LOP); therefore, it is unclear if similar acute effects would be observed if either a lower or higher restriction pressure is utilized. Additionally, LOP measurements were completed in the supine position; however, they were asked to perform exercise in a seated position. Sieljacks et al. (55) reported differences in LOP in the seated and supine positions, with seated LOP reportedly being significantly greater compared to supine LOP. As such, it is possible in the present investigation that the “effective” occlusion pressure utilized in the present investigation was lower than 80% LOP reported. While the authors do not believe this detracts from the results of the present investigation due to the repeated-measures crossover design, practitioners may want to consider utilizing a seated upright LOP as opposed to supine LOP when performing seated exercise. Additionally, the current investigation only utilized intermittent BFR, whereas previous investigations have also utilized continuous BFR. During continuous BFR, the restriction pressure is typically applied for the duration of exercise, including any recovery periods. The continuous restriction could thus have a significant effect on the physiological responses during and following exercise; however, this was beyond the scope of the current investigation. Resting metabolic rate is typically measured with a ventilated hood, which reduces leak risk and improves accuracy. In this study, we used a facemask breath-by-breath system to keep measurements consistent across rest, exercise, and recovery and to capture changes in oxygen use right after exercise. While this approach is common in EPOC research, small leaks around the mask cannot be completely ruled out (22). Another potential limitation of this study was the use of 140% VT for the heavy-intensity condition, which may have pushed some participants into the severe-intensity domain. However, the repeated-measures, crossover design likely minimized any impacts on the findings. Future studies may wish to determine both VT and the respiratory compensation point (RCP) to ensure exercise remains within the heavy-intensity domain. A further potential limitation of this study is the relatively small number of female participants. While combining male and female data offers broader insight, known sex differences in exercise-induced fat oxidation may have introduced variability to the results.

## Conclusion

In conclusion, this study attempted to examine the effects of BFR during low intensity cycling exercise on fat oxidation, EPOC, and excess energy expenditure. Results of the present investigation indicated that LI-BFR resulted in a significantly greater fat oxidation rate 60- and 120-min post-exercise compared to LI, and at 120 min post-exercise compared to HI. Additionally, EPOC was significantly greater in LI-BFR exercise compared to LI, but similar to HI exercise. Lastly, LI-BFR resulted in a significantly greater excess energy expenditure compared to both LI and HI exercise.

## Data Availability

The raw data supporting the conclusions of this article will be made available by the authors, without undue reservation.
